# Characteristics of infections with ancestral, Beta and Delta variants of SARS-CoV-2 in the PHIRST-C community cohort study, South Africa, 2020-2021

**DOI:** 10.1186/s12879-024-09209-z

**Published:** 2024-03-21

**Authors:** Cheryl Cohen, Jackie Kleynhans, Anne von Gottberg, Meredith L. McMorrow, Nicole Wolter, Jinal N. Bhiman, Jocelyn Moyes, Mignon du Plessis, Maimuna Carrim, Amelia Buys, Neil A. Martinson, Kathleen Kahn, Stephen Tollman, Limakatso Lebina, Floidy Wafawanaka, Jacques du Toit, Francesc Xavier Gómez-Olivé, Fatimah S. Dawood, Thulisa Mkhencele, for the PHIRST group, Stefano Tempia

**Affiliations:** 1 Centre for Respiratory Diseases and Meningitis, National Institute for Communicable Diseases of the National Health Laboratory Service, Johannesburg, South Africa; 2 School of Public Health, Faculty of Health Sciences, University of the Witwatersrand, Johannesburg, South Africa; 3 Centers for Disease Control and Prevention (CDC) COVID-19 Response, Atlanta, Georgia United States of America; 4 School of Pathology, Faculty of Health Sciences, University of the Witwatersrand, Johannesburg, South Africa; 5 Perinatal HIV Research Unit, University of the Witwatersrand, Johannesburg, South Africa; 6 DST/NRF Centre of Excellence for Biomedical Tuberculosis Research, University of the Witwatersrand, Johannesburg, South Africa; 7 Johns Hopkins University Center for TB Research, Baltimore, Maryland United States of America; 8 MRC/Wits Rural Public Health and Health Transitions Research Unit (Agincourt), Faculty of Health Sciences, School of Public Health, University of the Witwatersrand, Johannesburg, South Africa; 9 Africa Health Research Institute, Mtubatuba, KwaZulu-Natal South Africa

**Keywords:** SARS-CoV-2, South Africa, Epidemiology, Cohort study

## Abstract

**Background:**

Data on the characteristics of individuals with mild and asymptomatic infections with different SARS-CoV-2 variants are limited. We therefore compared the characteristics of individuals infected with ancestral, Beta and Delta SARS-CoV-2 variants in South Africa.

**Methods:**

We conducted a prospective cohort study in a rural and an urban site during July 2020-August 2021. Mid-turbinate nasal swabs were collected twice-weekly from household members irrespective of symptoms and tested for SARS-CoV-2 using real-time reverse transcription polymerase chain reaction (rRT-PCR). Differences in demographic and clinical characteristics, shedding and cycle threshold (Ct) value of infection episodes by variant were evaluated using multinomial regression. Overall and age-specific incidence rates of infection were compared by variant.

**Results:**

We included 1200 individuals from 222 households and 648 rRT-PCR-confirmed infection episodes (66, 10% ancestral, 260, 40% Beta, 322, 50% Delta). Symptomatic proportion was similar for ancestral (7, 11%), Beta (44, 17%), and Delta (46, 14%) infections (*p*=0.4). After accounting for previous infection, peak incidence shifted to younger age groups in successive waves (40-59 years ancestral, 19-39 years Beta, 13-18 years Delta). On multivariable analysis, compared to ancestral, Beta infection was more common in individuals aged 5-12 years (vs 19-39)(adjusted odds ratio (aOR) 2.6, 95% confidence interval (CI)1.1-6.6) and PCR cycle threshold (Ct) value <30 (vs >35)(aOR 3.2, 95%CI 1.3-7.9), while Delta was more common in individuals aged <5 (aOR 6.7, 95%CI1.4-31.2) and 5-12 years (aOR 6.6 95%CI2.6-16.7)(vs 19-39) and Ct value <30 (aOR 4.5, 95%CI 1.3-15.5) and 30-35 (aOR 6.0, 95%CI 2.3-15.7)(vs >35).

**Conclusions:**

Consecutive SARS-CoV-2 waves with Beta and Delta variants were associated with a shift to younger individuals. Beta and Delta infections were associated with higher peak viral loads, potentially increasing infectiousness.

**Supplementary Information:**

The online version contains supplementary material available at 10.1186/s12879-024-09209-z.

## Introduction

Understanding the epidemiology of infection caused by successive SARS-CoV-2 variants of concern (VOC) can provide insights into the reasons for the emergence and establishment of different VOCs [1]. SARS-CoV-2 VOCs have generally tended to emerge and rapidly replace previous circulating variants, making it challenging to separate changes in epidemiology as a result of increasing rates of prior immunity (due to infection or vaccination) in the population from intrinsic changes in virus transmissibility or pathogenesis.

While much is known about the epidemiology of different SARS-CoV-2 VOCs, most of these data focus on symptomatic infection and data from high-income countries predominate [2]. Less is known about the epidemiology of asymptomatic and mild infection and from sub-Saharan Africa. Data are also more limited on the epidemiology of the Beta VOC because of its more restricted geographic distribution [3]. Understanding the epidemiology of different VOCs can assist with public health planning for future emerging variants.

In a rural and an urban community in South Africa over 14 months from mid-July 2020 through end August 2021, we aimed to describe the timing of circulation and estimate the incidence rate by age group and site for different SARS-CoV-2 variants (ancestral, Beta, Delta) overall and accounting for immunity from previous infection or vaccination and compare the demographic and clinical characteristics, symptomatic fraction, duration of shedding, minimum PCR cycle threshold value (as a proxy for peak viral load) [4] and proportion previously infected, between different SARS-CoV-2 variants.

## Methods

We implemented a prospective household cohort study in a rural and an urban community in South Africa with twice weekly collection of mid-turbinate nasal swabs, symptom, and health-seeking data and serum collection every two months to measure SARS-CoV-2 antibodies. The Prospective Household cohort study of Influenza, Respiratory Syncytial virus and other respiratory pathogens community burden and Transmission dynamics in South Africa – COVID version (PHIRST-C) was based on a previous study (PHIRST) at the same sites from 2016-2018 [5, 6]. Detailed description of the study design and main results of this study have been published [7, 8]. Data included in this analysis are from the start of the study on 16 July 2020 and 27 July 2020 at the rural and urban site, respectively, to 28 August 2021, including 56 and 58 weeks of follow-up. Nasal swab collection began before the first wave peak in the district where the rural site was located and during the peak of the first wave at the urban site.

The rural site in Mpumalanga Province is nested within a health and socio-demographic surveillance system (HDSS) run by the Medical Research Council/University of Witwatersrand Rural Public Health and Health Transitions Research Unit, Agincourt [9, 10]. The urban site, Jouberton Township in the town of Klerksdorp, is located in the North West Province. Households were randomly selected, from the HDSS database in the rural site and using Global Positioning System (GPS) coordinates in the urban site. Households with >2 members and where ≥80% of eligible members consented to participate were eligible. Details on sample size, household selection, enrolment and data collection have been described previously [7, 8]. In brief, we first approached households previously enrolled in PHIRST, and then prospectively approached new potentially-eligible households using the site-specific sampling frame used for PHIRST until the required number of households were enrolled.

### Data collection

We collected individual baseline data, including demographics, HIV status, and history of underlying illness. Study staff visited participating households twice weekly (Monday-Wednesday and Thursday-Saturday) during July 2020-August 2021 to collect mid-turbinate nasal swabs from participants and information about symptoms, absenteeism, and health system contact. Data were entered by study staff in the field on tablets using REDCap (Research Electronic Data Capture) [11] and refresher training on specimen collection and the identification of respiratory signs and symptoms was conducted weekly. Blood specimens were collected from participants at enrolment (20 July–17 September 2020, blood draw (BD) 1), and every two months thereafter.

### Laboratory methods

Nasal specimens were collected using nasopharyngeal nylon flocked swabs, placed in Universal Transport Medium (UTM) and transported daily on ice packs to the National Institute for Communicable Diseases (NICD) in Johannesburg, South Africa, for testing. Nucleic acids were extracted from 200 µl of UTM using the Microlab NIMBUS Instrument (Hamilton, Nevada, USA) with the STARMag Universal Cartridge extraction kit (Seegene Inc., Seoul, Korea) according to manufacturer instructions. Specimens were tested for the presence of SARS-CoV-2 nucleic acids by rRT-PCR using the Allplex™ 2019-nCoV kit (Seegene Inc., Seoul, Korea) and a BioRad CFX96 thermal cycler, according to manufacturer instructions. From March 2021, samples were tested using the Allplex™ SARS-CoV-2/FluA/FluB/RSV kit (Seegene Inc., Seoul, Korea). A PCR cycle threshold (C_t_) value of <40 on ≥1 of 3 SARS-CoV-2 PCR targets (E/S, N and RdRp genes) was considered positive. All specimens testing rRT-PCR positive were confirmed by re-extraction of a second aliquot, and PCR testing in duplicate. Specimens testing positive on at least one duplicate rRT-PCR were considered positive. If a specimen was confirmed positive after re-extraction, the results [C_t_ value and targets testing positive] from the first positive test were included in the analysis. A lower C_t_ value on rRT-PCR (using the lowest C_t_value for any target during the episode) was used as a proxy for higher peak viral load, however formal quantification of viral RNA was not performed [4]. All confirmed positive samples were tested to identify variants of concern using the Allplex™ SARS-CoV-2 Variants I and Variants II assays (Seegene Inc., Seoul, Korea). The Variants I assay targets the RdRp gene, HV69/70 deletion, N501Y and E484K mutations and was therefore able to detect Alpha (B.1.1.7), Beta (B.1.351) and Gamma (P.1) variants. The Variants II kit detects the L452R, W152C, K417T and K417N mutations thus identifying the beta (B.1.351), Gamma (P.1) and Delta (B1.617.2) variants.

### Variant designation

For the assessment of variants, data from both the Allplex™ SARS-CoV-2 Variants I and II assay and sequencing were used.

Variants were determined based on:

1. At sample level, each sample was characterised using the Ion AmpliSeq SARS-CoV-2 Research Panel on the Ion Torrent Genexus Integrated Sequencer (ThermoFisher Scientific, USA) and assembled using the SARSCoV2 RECoVERY pipeline for whole genome sequencing and the Allplex™ SARS-CoV-2 Variants I and II PCR assays.
2. Variants were assigned firstly based on sequencing results where overall genome coverage was greater than 50% and where clade and lineage assignments (using the online Nextclade (https://clades.nextstrain.org/) and PANGOLin (https://pangolin.cog-uk.io/) applications were possible. This also enabled identification of known variants of concern as well as novel mutations.
3. Where sequence coverage was too low for classification, or where sequencing was not performed due to initial diagnostic rRT-PCR C_t_ values >35, variant rRT-PCR results were used. Where no variant-specific mutations were detected by the rRT-PCR, and the RdRp gene Ct was <35, and internal controls for both assays validated the integrity of the sample and the rRT-PCR result , the episodes were classified as attributed to ancestral virus. Where no variant-specific mutations were detected in the rRT-PCR, but the RdRp gene Ct was ≥35, the variant for the sample was designated as unassigned.
4. Any discrepancies identified between sequencing and rRT-PCR results were verified by re-extracting RNA from the original sample and repeating both sequencing and variant rRT-PCR assays. If results were still discrepant, the the sample was sequenced for a third time using a different aliquot.

A variant was allocated to each episode of infection according to the following hierarchical process:

1. At least one sample within the identified episode of infection with confirmed ancestral or variant result: ancestral or variant assigned to the entire episode.
2. No samples within the identified episode of infection with confirmed ancestral or variant result, but the episode is within a household cluster with at least one known episode of infection with confirmed ancestral or variant result: cluster ancestral or variant assigned to the episode.
3. No samples within the identified episode of infection with confirmed ancestral or variant result, and the episode is not within a household cluster with at least one known episode of infection with confirmed ancestral or variant result: ancestral or variant assigned to the episode based on wave as a proxy for lineage circulation (i.e., wave 1: ancestral; wave 2: Beta variant; wave 3: Delta variant).

Based on epidemic timing in the two communities, first wave (ancestral) episodes or clusters were defined as having onset before 19 December (week 51) of 2020 at both sites, second wave (Beta) as having onset before 9 June (week 20) 2021 in Agincourt and 23 June (week 23) 2021 in Klerksdorp, and third wave (Delta) as onset up to 28 August at both sites when intense follow-up ended.

### Serology

Serologic evidence of SARS-CoV-2 infection was determined using the Roche Elecsys® Anti-SARS-CoV-2 assay (Roche Diagnostics, Rotkreuz, Switzerland), using recombinant protein representing the nucleocapsid (N) antigen. The assay was performed on the Cobas e601 instrument, and a cut-off index of ≥1.0 was considered an indication of previous infection (seropositivity).

### HIV status determination

HIV status was obtained from patient-held medical records if a participant reported being HIV-infected, or by nurse-administered rapid HIV test with pre- and post-test counselling for participants with unknown, or self-reported HIV-negative status. Patients newly diagnosed with HIV were referred to the nearest primary health care facility for assessment and initiation of antiretroviral treatment.

### Vaccination status

Individuals were considered fully vaccinated against SARS-CoV-2 ≥14 days after they had received a single dose of the Johnson and Johnson Janssen (J&J) or two doses of the Pfizer BioNTech (Pfizer) vaccine and partially vaccinated if they had received any vaccine dose but not meeting the above criteria.

### Definitions and statistical analyses

We included individuals with ≥10 completed follow-up visits. We defined a SARS-CoV-2 serology-confirmed previously infected individual as at least one instance of SARS-CoV-2 antibody seropositivity. We defined a SARS-CoV-2 rRT-PCR-confirmed infection episode as at least one nasal swab rRT-PCR positive for SARS-CoV-2. For comparison of infection episodes by variant, only PCR-confirmed episodes were included. Infection episode duration was estimated from the first to the last day of SARS-CoV-2 rRT-PCR positivity. An illness episode was defined as an episode with ≥1 symptom reported from one visit before, to one visit after the SARS-CoV-2 infection episode. Asymptomatic infection episodes were defined as episodes where no symptoms were reported from one visit before, to one visit after the SARS-CoV-2 infection episode. Symptoms collected included fever (self-reported or measured tympanic temperature ≥38◦C), cough, difficulty breathing, sore throat, nasal congestion, vomiting, diarrhoea, abdominal pain or loss of smell or taste, muscle aches, fatigue, headache and confusion. Cumulative incidence was estimated as the total number of individuals experiencing at least one episode divided by the total number of individuals.

An rRT-PCR-confirmed household cluster was composed of all rRT-PCR-confirmed infection episodes within a household where the interval between the last rRT-PCR positive test of the first episode and the first rRT-PCR positive test of the second episode in any infection episode pair was not >14 days (representing >2 mean serial intervals) [8, 12]. This included clusters of an index case with no secondary cases. Previous infection was defined as previous rRT-PCR confirmed infection >28 days before or evidence of previous infection on serology.

We estimated incidence overall and for ancestral, Beta and Delta separately. We included only rRT-PCR confirmed infections, except for ancestral where we included all individuals who were seropositive at the start of follow-up in addition. Rates were expressed per person-year and were estimated from 5 March 2022, when the first case of SARS-CoV-2 was detected in South Africa until the end of follow-up for the individual. For estimation of incidence by subgroups (site and age groups), we divided the number of episodes in each subgroup by the number of person years of follow up by site and age group. For estimates of incidence adjusted for prior immunity, we used a similar approach but censored individuals following the first episode of infection with any variant. Specifically, individuals who had an episode of infection were censored from further follow-up for the study duration under the assumption that the episode had conferred immunity to subsequent infection.

For analysis comparing characteristics of infection episodes by variant, we only included episodes occurring with onset >14 days after the start of follow-up. This was because individuals tested positive at the start of follow-up (*n*=7 and *n*=32 at the rural and urban sites, respectively), and we did not know how long they had been shedding SARS-CoV-2 or if they had symptoms previously. Alpha variant infections were excluded because numbers were too few to allow meaningful comparison. Proportions were compared using the Chi-squared or Fisher’s exact test. We used multinomial logistic regression for the analysis of demographic and clinical characteristics, shedding and cycle threshold (C_t_) value of infection episodes with Beta and Delta variants vs ancestral. The ancestral strain was chosen as the reference group. Multinomial regression allows modelling of outcome variables with >2 categories (levels=j) and relates the probability of being in category j to the probability of being in a baseline category. A complete set of coefficients are estimated for each of the j levels compared with the baseline and the effect of each predictor in the model is measured as the relative risk ratio (RRR). We accounted for within-household clustering using random effects regression models. For the multivariable model, we considered all a-priori likely biologically associated factors with the outcome of interest for which we had available data. Variables included were site (urban, rural), age group in years (<5, 5-12, 13-18, 19-39, 40-59, ≥60), sex (female, male), HIV and viral load copies/ml (uninfected, infected <400, infected ≥400, HIV and/or viral load unknown), underlying illness other than HIV (present, absent), body mass index (underweight, normal weight, overweight, obese), symptoms (present, absent), shedding duration (≤4 days, >4 days), minimum Ct value (>35, 30-35, <30), previous infection (yes, no), SARS-CoV-2 vaccination status (unvaccinated, partially vaccinated, fully vaccinated). Variables were retained in the model using stepwise forward selection. Pairwise interactions were assessed graphically and by inclusion of product terms for all variables remaining in the final multivariable additive model. We conducted all statistical analyses using Stata version 14.1 (Stata Corp LP, College Station, Texas, USA). For each univariate analysis, we used all available case information. P values <0.05 were considered statistically significant.

### Ethics

The PHIRST protocol was reviewed and approved by the University of Witwatersrand Human Research Ethics Committee (Reference 150808) and amended to include enrolment and testing for COVID-19 on 24 June 2020. The study was registered on clinicaltrials.gov, registration number NCT02519803 11/08/2015 (https://clinicaltrials.gov/ct2/show/NCT02519803). Participants received grocery store vouchers of USD 3 per visit to compensate for the time required for specimen collection and interview. All participants provided informed consent. For minors informed consent was obtained from a parent or legal guardian.

## Results

**Fig. 1:**
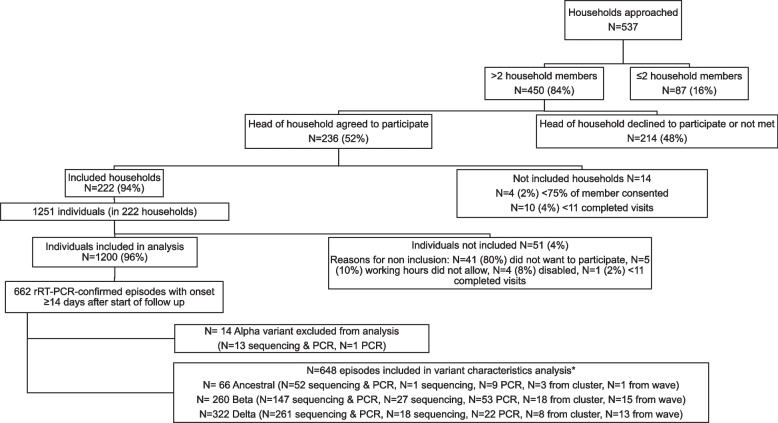
Flow chart of individuals included in the study, a rural and an urban site, South Africa, 2020-2021. PCR - polymerase chain reaction. * Sequencing and PCR - variant confirmed on whole genome sequencing and variant typing PCR, Sequencing - variant confirmed on sequencing only, PCR, variant confirmed on variant typing PCR only, from cluster - inferred from household cluster, from wave - inferred from wave timing

Characteristics of the study cohort have been described in detail previously [8]. Of 537 households approached, 450 (84%) had >2 household members, 236 (52% agreed to participate) and 222 (41%) were included in the analysis. Reasons for non-inclusion were <75% of household members consented (*n*=4, 2%) and participant completed <11 visits (*n*=10, 4%). There were 1251 eligible household members and 1200 (96%) were included (Fig. 1). The median number of household members in included households was 5 (interquartile range (IQR) 4-7), there was a median of 3 sleeping rooms (IQR 2-4), and 109 (49%) had ≥1 child aged <5 years, with a higher proportion in the rural community (Supplementary Table 1). Individuals from the rural community were younger, with a lower level of formal education and were more likely to be unemployed. HIV prevalence was similar at both sites (14% - 17%). At the end of follow up 5% (57/1200) of individuals were fully vaccinated against SARS-CoV-2.

**Fig. 2:**
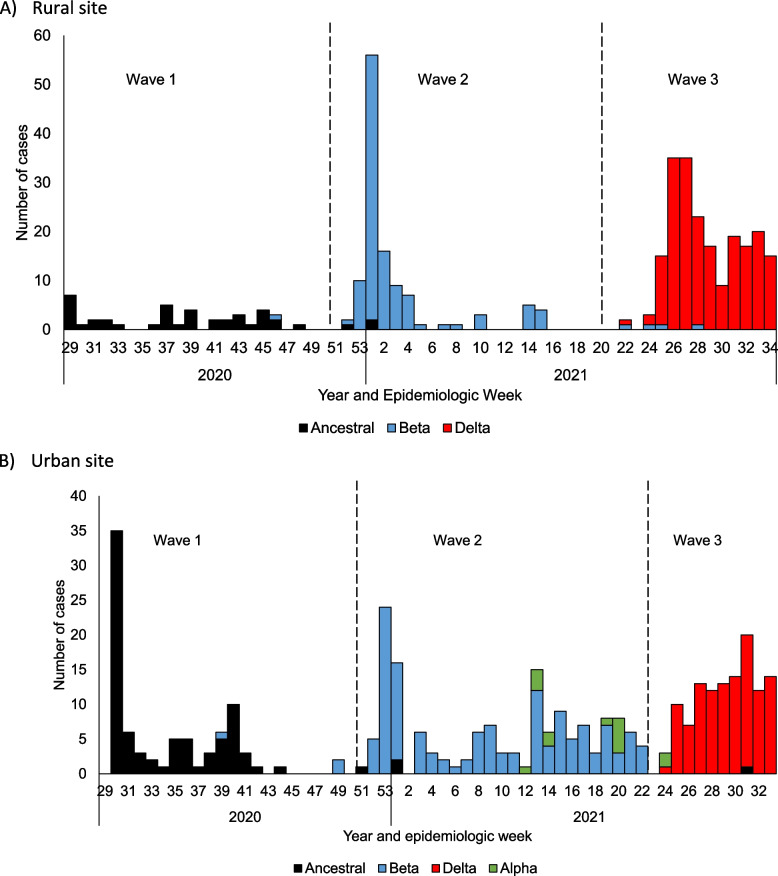
Epidemic curve of rRT-PCR-confirmed SARS-CoV-2 by non-variant or variant type at a rural and an urban site, South Africa, 2020-2021^*^. *Follow up began on 16 and 27 July 2020 at rural and urban site, respectively, vertical dashed lines indicates analysis cut off between first, second and third SARS-CoV-2 waves at each site, variant imputed for those without variant allocation

At the start of follow-up, ancestral SARS-CoV-2 was circulating in both communities and 1% (5/443) and 15% (73/498) of individuals with available data had serologic evidence of previous SARS-CoV-2 infection at the rural and the urban site, respectively (Fig. 2). The ancestral wave was followed by successive waves of Beta and Delta variant SARS-CoV-2 infection in both communities. Alpha variant circulated at low levels in the urban community.

During the follow-up period, there were 662 rRT-PCR-confirmed infection episodes with onset >14 days after the start of follow-up. Of these, 66 (10%) were ancestral, 260 (39%) Beta, 322 (49%) Delta and 14 (2%) Alpha variants. Alpha infections were excluded from further analysis because of low numbers. Symptomatic proportion was similar for ancestral (7, 11%), Beta (44, 17%), and Delta (46, 14%) infections (*p*=0.4) (Table 1). On multivariable analysis, compared to ancestral infection, infection with Beta was more common in individuals aged 5-12 years (vs 19-39 years) (adjusted odds ratio (aOR) 2.6, 95% confidence interval (CI) 1.1-6.6) and rRT-PCR Ct value <30 (vs >35) (aOR 3.2, 95% CI 1.3-7.9), while infection with Delta was more common in individuals aged <5 years (aOR 6.7, 95% CI 1.4-31.2) and 5-12 years (aOR 6.6 95% CI 2.6-16.7) (vs 19-39 years) and Ct value <30 (aOR 4.5, 95% CI 1.3-15.5) and 30-35 (aOR 6.0, 95% CI 2.3-15.7) (vs >35).

**Table 1:** Comparison of demographic and clinical characteristics, shedding and cycle threshold (Ct) value of infection episodes with Beta and Delta variants vs ancestral in a rural and an urban community, South Africa, 2020-2021

		Ancestral	Beta	Delta	Univariate Beta	Univariate Delta	Multivariable Beta	Multivariable Delta
Variable		n/N (%)	n/N (%)	n/N (%)	RRR^d^ (95% CI)		aRRR^d^ (95% CI)	
Site	Urban	38/66 (58)	141/260 (54)	116/322 (36)	0.9 (0.5-1.5)	0.4 (0.2-0.7)		
Age group (years)	<5	3/66 (5)	27/260 (10)	32/322 (10)	3.0 (0.8-10.8)	4.2 (1.2-15.2)	4.2 (0.9-19.7)	6.7 (1.4-31.2)
	5-12	9/66 (14)	59/260 (23)	113/322 (35)	2.2 (0.9-5.1)	5.0 (2.2-11.5)	2.6 (1.1-6.6)	6.6 (2.6-16.7)
	13-18	14/66 (21)	39/260 (15)	66/322 (21)	0.9 (.4-2.0)	1.9 (0.9-4.0)	0.8 (0.4-1.7)	1.5 (0.7-3.3)
	19-39	23/66 (35)	69/260 (27)	58/322 (18)	Reference	Reference	Reference	Reference
	40-59	12/66 (18)	38/260 (15)	32/322 (10)	1.1 (0.5-2.4)	1.1 (0.5-2.4)	0.9 (0.4-2.0)	0.9 (0.4-2.1)
	≥60	5/66 (8)	28/260 (11)	21/322 (7)	1.9 (0.6-5.4)	1.7 (0.6-4.9)	1.7 (0.6-5.0)	1.5 (0.5-4.6)
Sex	Female	44/66 (67)	166/260 (64)	185/322 (57)	0.9 (0.5-1.6)	0.7 (0.4-1.2)		
HIV and viral load copies/ml	Uninfected	52/66 (79)	200/260 (77)	270/322 (84)	Reference	Reference		
	Infected <400	7/66 (11)	35/260 (13)	26/322 (8)	1.3 (0.5-3.1)	0.7 (0.3-1.7)		
	Infected ≥400	3/66 (5)	14/260 (5)	7/322 ()2)	1.2 (0.3-4.4)	0.4 (0.1-1.8)		
	HIV and/or viral load unknown	4/66 (6)	11/260 (4)	19/322 (6)	0.7 (0.2-2.3)	0.9 (0.3-2.8)		
Other underlying illness^a^	Present	8/66 (12)	27/260 (10)	25/322 (8)	0.8 (0.4-1.9)	0.6 (0.3-1.4)		
BMI^b^	Underweight	2/66 (3)	18/260 (7)	32/320 (10)	2.2 (0.5-9.8)	2.6 (0.6-11.3)		
	Normal weight	29/66 (44)	121/260 (47)	181/320 (57)	Reference	Reference		
	Overweight	13/66 (20)	54/260 (21)	55/320 (17)	1.0 (0.5-2.1)	0.7 (0.3-1.4)		
	Obese	22/66 (33)	67/260 (26)	52/320 (16)	0.7 (0.4-1.4)	0.4 (0.2-0.7)		
Symptoms	Present	7/66 (11)	44/260 (17)	46/322 (14)	1.7 (0.7-4.0)	1.4 (0.6-3.3)		
Shedding duration	>4 days	48/66 (73)	208/260 (80)	255/322 (79)	1.5 (0.8-2.8)	1.4 (0.8-2.6)		
Minimum Ct value	>35	10/66 (15)	19/260 (7)	18/322 (6)	Reference	Reference	Reference	Reference
	30-35	6/66 (9)	6/260 (10)	36/322 (11)	2.3 (0.7-7.4)	3.3 (1.1-10.6)	2.8 (0.8-9.3)	4.5 (1.3-15.5)
	<30	50/66 (76)	215/260 (83)	268/322 (83)	2.3 (1.0-5.2)	3.0 (1.3-6.8)	3.2 (1.3-7.9)	6.0 (2.3-15.7)
Previous infection^c^		6/61 (10)	25/257 (10)	82/322 (25)	1.0 (0.4-2.5)	3.1 (1.3-7.5)	1.6 (0.6-4.3)	6.2 (2.3-16.4)
SARS-CoV-2 vaccine status before episode	Unvaccinated	62/66 (94)	237/260 (91)	297/322 (92)	Reference	Reference		
	Partially vaccinated	3/66 (5)	19/260 (7)	11/322 (3)	1.6 (05-5.8)	0.8 (0.2-2.8)		
	Fully vaccinated	1/66 (2)	4/260 (2)	14/322 (4)	1.0 (0.1-9.5)	2.9 (0.4-22.6)		

^a^Self-reported history of asthma, lung disease, heart disease, stroke, spinal cord injury, epilepsy, organ transplant, immunosuppressive therapy, organ transplantation, cancer, liver disease, renal disease or diabetes

^b^*BMI* Body mass index calculated using the formula (weight in kilograms)/(height in metres squared). We defined BMI categories as follows: underweight - age <18 years weight for age or BMI <-2 standard deviations of the World Health Organization (WHO) Child Growth Standards, age ≥18 years BMI <18.5kg/m2; overweight - age <18 years BMI >+1 and ≤+2 standard deviations of the WHO growth standards, age ≥18 years BMI ≥25 and <30kg/m2, obese – age <18 years BMI >+2 standard deviations of the WHO growth standards, age ≥18 years BMI ≥30 kg/m2

^c^on rRT-PCR or serology

^d^*aRRR* adjusted relative risk ratio estimated using multinomial logistic regression adjusted for clustering by site and household

**Fig. 3:**
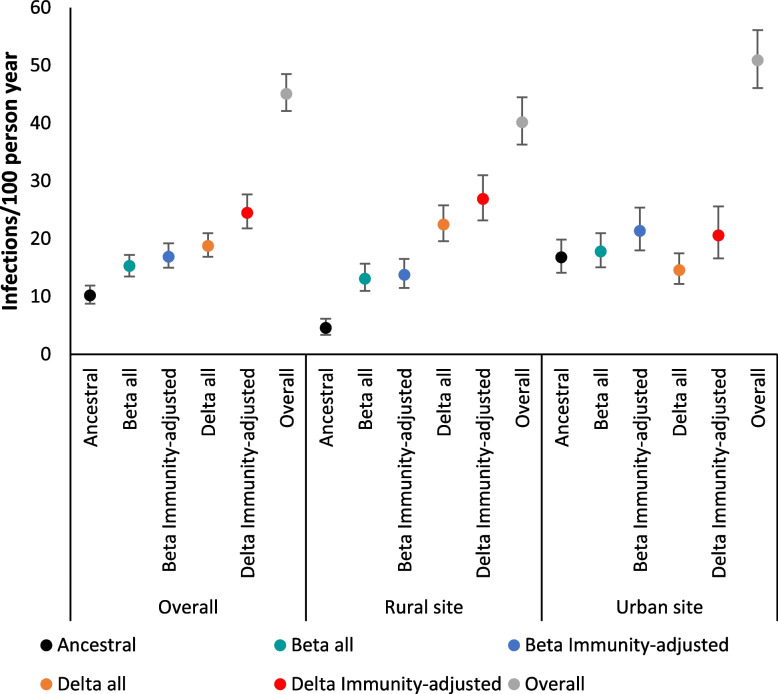
Rates^a^ of SARS-CoV-2 infections per 100 person years by SARS-CoV-2 variant and site, at a rural and an urban site, South Africa, 2020-2021. ^a^ Incidence rate estimated as number of episodes divided by the person time under observation

The incidence rate of SARS-CoV-2 infections for both sites combined was highest for the Delta variant (18.8 per 100 person-years, 95% CI 16.9-21.0), followed by Beta (15.3 per 100 person-years, 95% CI 13.5-17.2) and ancestral (10.2 per 100 person-years, 95% CI 8.8-11.9) (Table 2). This differed by site with the rural site experiencing the highest rates for Delta followed by ancestral-1 and Beta, while the urban site experienced the highest rate for Beta followed by ancestral-1 and Delta (Fig. 3). The overall rate of rRT-PCR-confirmed infections was higher at the urban site (50.9 per 100 person-years 95% CI 46.1-56.1 vs 40.2 95% CI 36.3-44.5).

**Table 2:** Rates^a^ of SARS-CoV-2 infections per 100 person years by SARS-CoV-2 variant and site, at a rural and an urban site, South Africa, 2020-2021

Site	Person years of follow up overall	All variants Rate (95% CI)	Wuhan-Hu Rate (95% CI)	Beta Rate (95% CI)		Delta Rate (95% CI)	
		Overall	Overall	Overall	Immunity-adjusted	Overall	Immunity-adjusted
Overall	1709.7	45.1 (42.1-48.5)	10.2 (8.8-11.9)	15.3 (13.5-17.2)	16.9 (15.0-19.2)	18.8 (16.9-21.0)	24.5 (21.8-27.7)
Rural site	916.3	40.2 (36.3-44.5)	4.6 (3.4-6.2)	13.1 (11.0-15.7)	13.8 (11.5-16.5)	22.5 (19.6-25.8)	26.9 (23.2-31.0)
Urban site	793.5	50.9 (46.1-56.1)	16.8 (14.1-19.9)	17.8 (15.1-21.0)	21.4 (18.0-25.4)	14.6 (12.2-17.5)	20.6 (16.6-25.6)

^a^Incidence rate estimated as number of episodes divided by the person time under observation

**Fig. 4:**
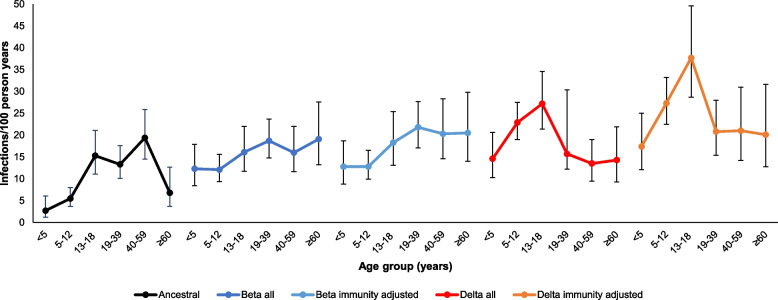
Rates of SARS-CoV-2 infections per 100 person years by age group and variant, at a rural and an urban site, South Africa, 2020-2021^a^. ^a^Rates for Beta and Delta variants are presented first for the entire population (all) and second after removing individuals with previous infection or vaccination from the population (immunity adjusted). Bars represent 95% confidence intervals around rate estimates.

Age-specific incidence rates varied by SARS-CoV-2 variant (Fig. 4 and Supplementary Table 2). For ancestral the highest incidence was in individuals aged 40-59 years, followed by 13-18 years and 19-39 years. Peak incidence for Beta variant infection was among those ≥60 years followed by 19-39 years, while peak incidence for Delta infection was in 13-18 years followed by 5-12 years. After accounting for previous infection in different age groups, peak incidence showed a shift to younger age groups in successive waves, from 40-59 years with ancestral to 19-39 years for Beta and 13-18 years with Delta variant infections.

## Discussion

In this study, comparing the epidemiology of intensively ascertained mild and asymptomatic infections in a rural and urban South African cohort, we found that infection with Beta and Delta variants was more common in younger individuals compared to ancestral. After accounting for immunity due to previous infection, the peak age of infection shifted to younger age groups with each successive wave. Beta and Delta variant infections were also associated with lower minimum Ct values (a proxy for higher peak viral load) suggesting the potential for increased transmissibility. These shifts may contribute to the increased fitness of successive emerging viral variants and replacement of previous variants.

Several studies have described a shift to increased cases among children and adolescents with the Delta variant but the relative contribution of previous immunity and relaxation of restrictions such as school closures vs intrinsic characteristics of the VOC remains unclear [13, 14]. Data on the age distribution of individuals infected with the Beta variant are more limited because of its limited geographic distribution. A South African surveillance study found an increased incidence of diagnosed cases among individuals aged 1-18 years with the Beta variant compared to ancestral, suggesting a possible younger age distribution with this variant [15]. The same study also found increased incidence with Delta compared to Beta in this age group. A limitation of this study was the inclusion of routinely diagnosed cases which is highly affected by testing practices and availability as <10% of SARS-CoV-2 cases in South Africa are diagnosed [7]. In this intensively tested cohort, we show that with near-complete infection ascertainment and after accounting for immunity conferred by previous infection, the age experiencing the highest incidence decreased with each successive wave of infection. Our findings may not, however, be generalisable to other settings given differential contact rates and likely different rates of case ascertainment. Although we could account for effects of previous infection and immunity, we were not able to adjust for the effects of progressive relaxation of restrictions and opening of schools which could have in part driven the shifts to younger age groups. Interestingly, despite the decrease in the peak age group with successive waves, infection incidence was lowest among children aged <5 years in each wave. A major limitation of our study is that we do not have data from the subsequent Omicron BA.1 wave to evaluate whether this trend continued with eventual peak infection incidence moving to primary school children and toddlers as has been described for several seasonal respiratory viruses [6, 16].

Several previous studies have described a lower Ct value (proxy of higher viral load) for Beta and Delta infections [17, 18]. We have also previously described that lower minimum Ct values are associated with increased likelihood of symptoms [8]. The majority of these studies included symptomatic patients, a strength of our study is the inclusion of mostly mild and asymptomatic cases, representative of the majority of infections in South Africa. The lower Ct values likely contribute to increased transmissibility of emerging VOCs increasing viral fitness in relation to ancestral [19, 20].

Interestingly we did not observe differences in the symptomatic fraction of individuals infected with different VOCs. Several previous studies have described increased severity of infections with Beta and Delta [3, 21]. Possible reasons for no observed difference could be low power because of the low symptomatic fraction or the fact that differences in severity manifest more strongly at the severe end of the spectrum. The symptomatic fraction in our study was approximately 15%, substantially lower than that of a large systematic review, which estimated symptomatic fraction of 80% [22]. Other studies in Africa have also suggested that the symptomatic fraction may have been lower in some African settings compared to other settings [23, 24]. Possible reasons for the lower observed symptomatic fraction could include the study design with frequent sampling irrespective of symptoms which may allow detection of transient infections which might not otherwise be recorded or younger age distribution of the population. It is unlikely that individuals were pre-symptomatic but would have developed symptoms later because participants were followed up for 13 months with twice-weekly symptom questionnaires.

We observed higher attack rates in the urban compared to the rural site, perhaps due to more intense contact in this crowded setting or due to chance. We also observed different attack rates by variant and wave in the two sites indicating the heterogeneity of transmission in different geographic areas within a single country. In the urban site, the highest incidence was with Delta but in the rural site, the highest incidence was with Beta. Possible reasons for this difference include the effects of previous immunity as the rural site experienced a very small first wave or different mixing patterns. The Beta wave coincided with the Christmas and New Year period when large numbers of people travel to rural areas potentially driving increased transmission in these communities.

Our study had some limitations including low numbers of infections with the different VOC potentially limiting the ability to detect associations. We also had low numbers of individuals in some age groups limiting power to detect associations.The period of follow-up ended before the emergence of the Omicron VOC, meaning that we are unable to provide data on Omicron. We did not collect data on individual behaviours which could have increased infection risks such as mask use or school attendance, limiting our ability to evaluate the impact of these on rates of infection. We did not quantify viral RNA load but instead this was inferred using Ct values as proxy. It is possible that some infections were missed on rRT-PCR; however, a previous analysis of the same dataset found a high correlation between infections confirmed on rRT-PCR and serology, with 90.4% of infections identified on serology also confirmed on rRT-PCR and 90.8% of individuals who were rRT-PCR positive seroconverting after the episode [8].

In conclusion, we provide a detailed description of the epidemiology and incidence of infection with different SARS-CoV-2 variants, ascertained through intensive systematic testing, allowing almost complete infection ascertainment. We found that after accounting for previous immunity, successive waves of infection with the Beta and Delta VOCs were associated with shifts to younger peak age of infection. We also described lower minimum Ct values in Beta and Delta VOC infections suggesting higher peak viral load and potential increased infectivity. While these variants are no longer circulating globally, the detailed study of the epidemiology of successive viral variants provides helpful insights into the changing epidemiology of a newly emerged viral pathogen which may inform understanding in future pandemics.

### Supplementary Information

**Supplementary Material 1.**

## Data Availability

The investigators welcome enquiries about possible collaborations and requests for access to the data set*.* Data (including individual participant data and a data dictionary defining each field in the set) will be shared after approval of a proposal and with a signed data access agreement. Investigators interested in more details about this study, or in accessing these resources, should contact the principle investigator, Prof Cheryl Cohen, at NICD (cherylc@nicd.ac.za).
