# Supplementary material for: Characteristics of infections with ancestral, Beta and Delta variants of SARS-CoV-2 in the PHIRST-C community cohort study, South Africa, 2020-2021
Source: BMC Infect Dis. 2024 Mar 21;24:336. doi: 10.1186/s12879-024-09209-z (PMC10956206; doi:10.1186/s12879-024-09209-z)
Supplement: Supplementary file 1 — Supplementary Material 1. [file 12879_2024_9209_MOESM1_ESM.docx]

Supplementary table 1: Baseline characteristics of households and individuals included in a Prospective Household cohort study of Influenza, Respiratory Syncytial virus and other respiratory pathogens community burden and Transmission dynamics in South Africa – COVID version (PHIRST-C) in a rural and an urban community, South Africa, 2020-2021

| **Characteristic** | **Overall**  **n (%) or median (IQR)** | **Rural**  **n (%) or median (IQR)** | **Urban**  **n (%) or median (IQR)** |
| --- | --- | --- | --- |
| **Household level characteristics** | **N=222** | **N=114** | **N=108** |
| Number of household members  3-5  6-10  >10 | 131 (59)  82 (37)  9 (4) | 62 (54)  48 (42)  4 (4) | 69 (60)  34 (31)  5 (5) |
| Median number of household members | 5 (4-7) | 5 (4-7) | 5 (4-6) |
| Number of rooms  1-4  5-9  ≥10 | 76 (34)  138 (62)  8 (4) | 38 (33)  69 (61)  7 (6) | 38 (35)  69 (64)  1 (1) |
| Median number of rooms | 5 (4-7) | 5 (3-8) | 5 (4-6) |
| Number of rooms for sleeping  1-2  3-4  >4 | 93 (42)  106 (48)  23 (10) | 37 (32)  57 (50)  20 (18) | 56 (52)  49 (45)  3 (3) |
| Median number of rooms for sleeping | 3 (2-4) | 3 (2-4) | 2 (2-3) |
| Crowding (>2 people/sleeping room) | 83 (37) | 38 (33) | 45 (42) |
| Child aged <5 years in house | 109 (49) | 67 (59) | 42 (39) |
| Household member smokes indoors | 59 (27) | 13 (11) | 46 (43) |
| Main water source tap inside (vs tap outside) | 135 (61) | 58 (51) | 77 (71) |
| Handwashing place with water in house | 207 (93) | 103 (90) | 104 (96) |
| Main fuel for cooking  Electricity  Wood  Paraffin/gas/other | 141 (64)  79 (36)  2 (1) | 35 (31)  79 (69)  0 (0) | 106 (98)  0 (0)  2 (2) |
| Monthly household income^a^  ≤R800 (<USD54)  R801-R1600 (USD55-108)  R1601-R3200 (USD109-116)  R3201-R6400 (USD117-232)  R6401-R12800 (USD233-464)  >R12800 (>USD464) | 12 (6)  38 (18)  90 (43)  54 (26)  11 (5)  3 (1) | 8 (7)  15 (13)  48 (42)  33 (29)  9 (8)  1 (1) | 4 (4)  23 (24)  42 (45)  21 (22)  2 (2)  2 (2) |
| **Individual level characteristics** | **N=1200** | **N=643** | **N=557** |
| Age group (years)  <5  5-12  13-18  19-39  40-59  ≥60 | 154 (13)  340 (28)  170 (14)  265 (22)  168 (14)  103 (9) | 99 (15)  211 (33)  88 (14)  131 (20)  68 (11)  46 (7) | 55 (10)  129 (23)  82 (15)  134 (24)  100 (18)  57 (10) |
| Female sex | 717 (60) | 409 (64) | 308 (55) |
| Level of education^b^  No schooling  Primary schooling  Some secondary  Secondary completed  Post-secondary | 59 (11)  89 (17)  230 (43)  142 (27)  15 (3) | 35 (14)  44 (18)  83 (34)  80 (33)  3 (1) | 24 (8)  45 (16)  147 (51)  62 (21)  12 (4) |
| Employment^b^  Unemployed  Employed  Student  Pensioner | 352 (66)  27 (5)  109 (20)  47 (9) | 171 (70)  10 (4)  38 (16)  26 (11) | 181 (62)  17 (6)  71 (24)  21 (7) |
| Reported alcohol use^c^ | 196 (30) | 37 (12) | 159 (47) |
| Reported current cigarette smoking^c^ | 124 (19) | 12 (4) | 112 (33) |
| HIV status^d^  Uninfected  Infected  Unknown | 971 (85)  176 (15)  53 | 520 (86)  84 (14)  39 | 451 (83)  92 (17)  14 |
| HIV viral load^e^  ≥400 copies/ml | 31 (19) | 11 (14) | 20 (21) |
| CD4+ T cell count^f^  <200/ml | 14 (8) | 5 (6) | 9 (11) |
| Previous tuberculosis | 40 (3) | 11 (2) | 29(5) |
| Current tuberculosis | 5 (<1) | 1 (<1) | 4 (1) |
| Other underlying illness^g^ | 125 (10) | 42 (7) | 83 (15) |
| Influenza vaccination 2020 | 22 (2) | 18 (3) | 4 (1) |
| Influenza vaccination 2021 | 7 (1) | 6 (1) | 1 (<1) |
| Fully vaccinated against SARS-CoV-2 vaccine by end of follow up^l^ | 57 (5) | 23 (4) | 34 (6) |
| Pneumococcal vaccine up to date for age^j^  Yes  No  No data | 109 (92)  9 (8)  36 | 73 (97)  2 (3)  24 | 36 (84)  7 (16)  12 |
| DTaP-IPV/Hib vaccine up to date for age^i^  Yes  No  No data | 113 (96)  5 (4)  36 | 75 (100)  0 (0)  24 | 38 (88)  5 (12)  12 |

HIV – Human immunodeficiency virus, DTaP-IPV/Hib – Diphtheria, tetanus, acellular pertussis, inactivated polio, *Haemophilus influenzae* type B vaccine, IQR – interquartile range, OR – odds ratio, CI – confidence interval, NE – not estimated, n - number, USD – United States Dollar. Penalized logistic regression used for cells with zero values.

^a^Data available for 208 households, 114 rural and 94 urban ^b^Individuals aged >18 years with available data N=535, 245 at rural site and 290 at urban site ^c^Individuals aged ≥15 years N=643, 303 at rural site and 340 at urban site ^d^% and p value among individuals with known status ^e^Among 176 PLHIV, 166 (94%) reported currently receiving antiretroviral treatment (ART), of 165 PLHIV with data on CD4+ T cell count, 151 (92%) were >200 /ml, of 166 individuals with viral load data, 136 (82%) had <400 copies/ml ^g^Self-reported history of asthma, lung disease, heart disease, stroke, spinal cord injury, epilepsy, organ transplant, immunosuppressive therapy, organ transplantation, cancer, liver disease, renal disease or diabetes ^j^Individuals aged <5 years N=154, 99 at rural site and 55 at urban site, 118 with available vaccination data, 75 at the rural site and 43 at the urban site ^k^Estimated using logistic regression adjusted for clustering by site and household ^l^ Of 57 individuals who were fully vaccinated by the end of follow up, 23 received a single dose of the Johnson and Johnson vaccine and 34 received 2 doses of Pfizer vaccine and 47 (82%) were vaccinated from June through September. An additional 58 individuals received the first dose of Pfizer vaccine during the follow up period.

Supplementary table 2: Rates^a^ of SARS-CoV-2 infections per 100 person years by SARS-CoV-2 variant and age group, at a rural and an urban site, South Africa, 2020-2021

| **Age group (years)** | **Person years of follow up** | **All variants**  **Rate (95% CI)** | **Ancestral**  **Rate (95% CI)** | **Beta**  **Rate (95% CI)** | **Rate (95% CI)** | **Delta**  **Rate (95% CI)** | **Rate (95% CI)** |
| --- | --- | --- | --- | --- | --- | --- | --- |
|  |  | **Overall** | **Overall** | **Overall** | **Immunity-adjusted** | **Overall** | **Immunity-adjusted** |
| <5 | 219.7 | 31.0 (24.4-39.3) | 2.7 (1.2-6.1) | 12.3 (8.4-17.9) | 12.8 (8.8-18.7) | 14.6 (10.3-20.6) | 17.4 (12.1-25.0) |
| 5-12 | 494.5 | 41.1 (35.8-47.1) | 5.5 (3.7-8.0) | 12.1 (9.4-15.6) | 12.8 (9.9-16.5) | 22.9 (19.0-27.5) | 27.3 (22.5-33.3) |
| 13-18 | 242.6 | 60.6 (51.5-71.2) | 15.3 (11.1-21.1) | 16.1 (11.7-22.0) | 18.3 (13.1-25.4) | 27.2 (21.4-34.6) | 37.7 (28.7-49.6) |
| 19-39 | 368.4 | 48.3 (41.7-56.0) | 13.3 (10.1-17.6) | 18.7 (15.0-23.7) | 21.8 (17.1-27.7) | 15.7 (12.2-20.4) | 20.8 (15.4-28.0) |
| 40-59 | 237.6 | 49.2 (41.1-59.0) | 19.4 (14.5-25.9) | 16.0 (11.6-22.0) | 20.3 (15.6-28.3) | 13.5 (9.5-19.0) | 21.0 (14.2-31.0) |
| ≥60 | 146.8 | 40.2 (31.1-51.9) | 6.8 (3.7-12.7) | 19.1 (13.2-27.6) | 20.5 (14.0-29.8) | 14.3 (9.3-21.9) | 20.1 (12.8-31.6) |

^a^ Incidence rate estimated as number of episodes divided by the person time under observation
